# Caveolae: A Role in Endothelial Inflammation and Mechanotransduction?

**DOI:** 10.3389/fphys.2016.00628

**Published:** 2016-12-20

**Authors:** Waled A. Shihata, Danielle L. Michell, Karen L. Andrews, Jaye P. F. Chin-Dusting

**Affiliations:** ^1^Cardiovascular Disease Program and Department of Pharmacology, Biomedical Discovery Institute, Monash UniversityClayton, VIC, Australia; ^2^Vascular Pharmacology, Baker IDI Heart and Diabetes InstituteMelbourne, VIC, Australia

**Keywords:** blood pressure, inflammation, endothelial cells, haemodynamics, caveolae (or caveolin-1), mechanotransduction

## Abstract

Vascular inflammation and disease progression, such as atherosclerosis, are in part a consequence of haemodynamic forces generated by changes in blood flow. The haemodynamic forces, such as shear stress or stretch, interact with vascular endothelial cells, which transduce the mechanical stimuli into biochemical signals via mechanosensors, which can induce an upregulation in pathways involved in inflammatory signaling. However, it is unclear how these mechanosensors respond to shear stress and most significantly what cellular mechanisms are involved in sensing the haemodynamic stimuli. This review explores the transition from shear forces, stretch and pressure to endothelial inflammation and the process of mechanotransduction, specifically highlighting evidence to suggest that caveolae play as a role as mechanosensors.

Cardiovascular disease (CVD) is the primary cause of death globally, accounting for over 17 million deaths worldwide in 2008 (WHO, 2013). By 2030, it is predicted that ~25 million people will die annually from cardiovascular-related disorders. Cardiovascular diseases encompass all disease involving heart and blood vessel defects, such as coronary heart disease, myocardial infarction, and cerebrovascular disease (including stroke) as well as a number of other fibrotic disorders. Importantly, increased exposure to CVD risk factors such as smoking, alcohol abuse, physical inactivity, and high cholesterol (hypercholesterolemia) contribute to the growing CVD mortality rates in the western world (WHO, 2013). Of note, elevated blood pressure (BP) or hypertension contributes to more deaths and disease globally than any other risk factor (Forouzanfar et al., 2015).

Elevated systolic and/or diastolic blood pressure, resulting from increased systemic vascular resistance or cardiac output, exerts a complex mechanical insult on the arterial wall. Mechanical forces generated at the endothelium in the form of shear stress due to blood flow and stretch caused by blood pressure can potently affect vascular tone resulting in changes in morphology, function, gene expression and the release of vasoactive endothelial autacoids. Several different mechanosensors and multiple signaling pathways have been proposed as regulators of the endothelium's response. There is some evidence that integrins, the cytoskeleton, ion channels, and G proteins may be activated by stretch in vascular smooth muscle cells and that exertion of circumferential stretch on endothelial cells results in increased ICAM-1 (Cheng et al., 1996), VCAM-1 and E-selectin expression (Yun et al., 1999), which are hallmarks of endothelial inflammation. More recently, caveolae, 50- to 100-nm flask-shaped invaginations of the plasma membrane, have also been implicated as mechanosensors. Therefore, it is not surprising that chronic increases in BP have also been shown to result in vascular inflammation where reduced nitric oxide (NO) bioavailability is thought to contribute to endothelial dysfunction, increased reactive oxygen species (ROS) and an upregulation in the production of inflammatory cytokines and chemokines (Dalekos et al., 1997; Chae et al., 2001; Fernandez-Real et al., 2001; Bautista et al., 2004; Zhao et al., 2004; Lieb et al., 2008; Yu et al., 2010).

## Endothelial dysfunction

The endothelium plays a crucial role in the modulation of blood vessel function via its ability to regulate vascular tone, inflammation and haemostasis. Vascular tone is regulated via the release of constrictors (such as angiotensin II and endothelin 1) and dilators (including NO and certain arachidonic acid metabolites such as prostacyclin), which mediate the contraction and relaxation of the underlying smooth muscle cells (for review see, Savoia et al., 2011). Nitric oxide, a potent vasodilator, regulates endothelial function via its depressor, anti-hypertrophic and anti-inflammatory effects by direct inhibition of inflammatory signaling pathways. Endothelial dysfunction is characterized by impaired endothelium-dependent vasodilation, usually due to the loss of NO and an overall imbalance between vasoconstrictors and vasodilators, which leads to activation and inflammation of the endothelium. This reduced NO bioavailability is damaging given its role in inhibiting low-density lipoproteins (LDL) oxidation, which forms the basis of plaque development seen in atherosclerosis (Jessup et al., 1992; Yates et al., 1992; Malo-Ranta et al., 1994; Rubbo et al., 1995; Maccarrone et al., 1996; Goss et al., 1997). Accordingly, endothelial dysfunction can be triggered by diverse disturbances such as increased levels of LDL cholesterol, endotoxin and smoking. Endothelial dysfunction is one of the first clinical correlates of hypertension and atherosclerosis and its presence is a predictor of future cardiovascular events (Gokce et al., 2002). Endothelial dysfunction is also associated with increased oxidative stress which stimulates the inflammatory signaling pathways, involved in ROS production (Savoia et al., 2011). A consequence of increased ROS production and activity is the subsequent increase in NO catabolism, contributing to reduce NO bioavailability. This imbalance in NO-ROS can result in the expression of genes involved in inflammation and upregulation of inflammatory proteins.

**Figure 1:**
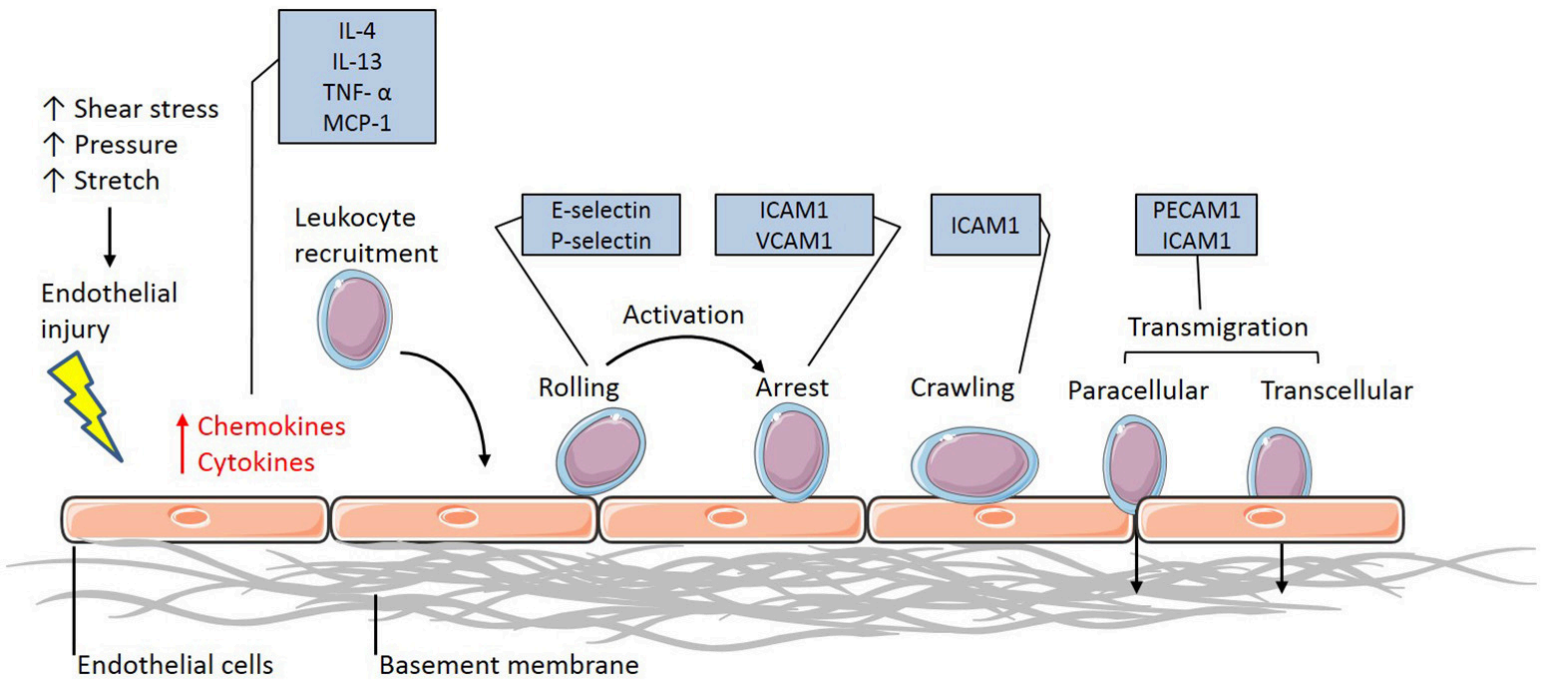
**The leukocyte adhesion cascade**. The leukocyte adhesion cascade describes the process involving complex leukocyte to endothelium interactions. It involves the activation of the endothelium, which upregulates inflammatory signaling mechanisms through the release of inflammatory cytokines and chemokines. Circulating leukocytes are then recruited to the activated endothelium, where they firmly adhere via various classes of adhesion molecules. Once the leukocytes have reached an arrest phase, they crawl until they reach complete arrest and are able to transmigrate through the vessel wall and differentiate into macrophages (IL-4, Interleukin-4; IL-13, Interleukin-13; TNF-α, Tumor necrosis factor-alpha; MCP-1, Monocyte chemoattractant protein-1; ICAM-1, Intercellular cell adhesion molecule-1; VCAM-1, Vascular cell adhesion molecule-1; PECAM-1, Platelet endothelial cell adhesion molecule-1).

Vascular inflammation requires the activation of the endothelium to initiate the interaction between the circulating immune cells and the endothelium. Once activated, the endothelium releases the inflammatory cytokines interleukin-4 (IL-4) and 13 (IL-13) in humans and tumor necrosis factor α (TNFα), IL-1, lipopolysaccharide (LPS) and IL-4 in murine populations (Yao et al., 1996; Pan et al., 1998; Woltmann et al., 2000), which signal to and attract leukocytes to the site of injury (Figure 1). Although this allows the immune cells to flow in close proximity to the endothelial layer, rolling and adhesion to the endothelial layer only occurs in the presence of adhesion molecule expression. The upregulation of surface adhesion molecules, including E-selectin and P-selectin, allow for the initial intimate interactions between the flowing leukocytes and endothelium, effectively recruiting the immune cells (Figure 1). Further upregulation in the expression of adhesion molecules such as intercellular and vascular cell adhesion molecules (ICAM-1 and VCAM-1, respectively) in the endothelium of atherosclerotic-prone vasculature (Figure 1) promotes adhesion between the monocyte and endothelium, interacting with integrins expressed at the surface of the monocytes. These integrins consist of α- and β-subunits and exist in an inactive, low affinity binding state prior to activation. Once activated by chemokines released by the leukocyte, the α/β-integrins undergo a conformational change allowing for high-affinity binding to ICAM-1 and VCAM-1 to occur (Figure 1). The integrin-CAM complex results in an increased leukocyte-endothelium adhesion, consequently halting leukocyte rolling and allowing for arrest to occur (Figure 1). Once the endothelial adhesion molecules bind to the cells, via L-selectin, expressed on the surface of leukocytes, the process of disease pathogenesis begins (Eriksson et al., 2001).

Importantly, it is well-recognized that some vascular beds are more susceptible to the development of atherosclerosis than others due to differences in flow parameters and haemodynamic forces. Vascular beds which are subjected to turbulence and oscillating flow or pathological shear stress are more prone to the development of atherosclerotic plaques, compared to sites of laminar flow (Davies et al., 2013). Indeed, it has been shown that exposure to haemodynamic forces results in an upregulated inflammatory response, observed by increased endothelial adhesion molecule expression, which in turn promotes the process of leukocyte rolling and adhesion, essentially driving the development of atherosclerosis (Morigi et al., 1995; Sampath et al., 1995; Chiu et al., 2004). Of note, disease pathogenesis is highly dependent on the nature of haemodynamic force generated and the location in which it occurs, as alluded to earlier.

## Haemodynamic forces

Under basal conditions, fluid shear stress and circumferential stretch results in continuous release of compensatory vasoactive substances to maintain homeostatic balance. Circumferential wall stretch is due to the pulsatile luminal pressure exerted on the vascular smooth muscle cells whereas shear stress is an effect of the frictional force created by the flow of blood along the endothelial cells. While there are studies demonstrating the influence of stretch on endothelial cells (von Offenberg Sweeney et al., 2004; Tian et al., 2016), shear stress is thought to be the prominent mechanical force exerted. The magnitude of endothelial shear stress in vessels is dependent on the velocity of blood flow, direction, and obstructions along the vessel, as well as the location of flow in the vascular tree.

Under physiological conditions, the flow of blood along straight vessels, also known as undisturbed laminar flow, results in physiological shear stress values of between 15 and 70 dyn/cm^2^, which is thought to promote a more cardioprotective phenotype (Dardik et al., 2005). Indeed, cultured endothelial cells from different human vessels demonstrate reduced inflammation following exposure to physiological shear stress, similar to that of laminar flow, compared to static conditions (Luu et al., 2010). Physiological shear stress conditions have also been shown to lead to anti**-**inflammatory effects, with reduced TNFα**-**induced adhesion molecule expression (Yamawaki et al., 2003). Yet conflicting reports do exist. A study using MRI technology has shown that one patient was found to develop plaque ulceration at the location with the highest shear stress (Groen et al., 2008). This finding suggests that physiological levels of shear stress may not be protective in areas of vulnerable plaques. However, it is well-established that sites in the vascular tree most vulnerable to atherosclerotic plaques include the inner curve of vessels, as well as vessel bifurcations and branches. At these sites, disturbed laminar flow is at its most prominent and presents in two forms: Unidirectional, which results in pathological shear stress with low shear values (<12 dyn/cm^2^) or bidirectional turbulent flow. Pathological levels of shear stress have been found to be associated with advanced inflammation observed by decreased NO bioavailability, upregulated LDL oxidation, degradation of extracellular matrix and apoptosis (Hwang et al., 2003; Gambillara et al., 2006; Chatterjee et al., 2012). This, in combination with a promotion in oxidative stress, results in homeostatic imbalance, which consequently leads to both vascular and plaque remodeling, contributing to an overall worsened cardiovascular outcome(Dardik et al., 2005; Cheng et al., 2006). Recently, the concept of shear stress induced signaling has been advanced with findings that endothelium can sense differences in the frequency spectrum (as a result of pulsatile flow) of shear and it is this that influences the activation of inflammatory signaling pathways (Feaver et al., 2013). The onset in the abovementioned pathophysiological processes is detected by mechanosensors, triggered by shear which leads to endoplasmic reticulum stress and activation of inflammatory pathways such as nuclear factor kappa B (NFκB), c-Jun N-terminal kinase (JNK), p21-activated kinase (PAK), and ROS as well as a dampening of anti-inflammatory processes such as eNOS (Nagel et al., 1999). Activation of these pathways can lead to upregulation in endothelin-1 expression, secretion of pro-inflammatory cytokines and increased expression of adhesion molecules such as ICAM-1, VCAM-1, monocyte chemoattractant protein-1 (MCP-1) and E-selectin (Walpola et al., 1995; Chiu et al., 2004).

## Mechanosensors

**Figure 2:**
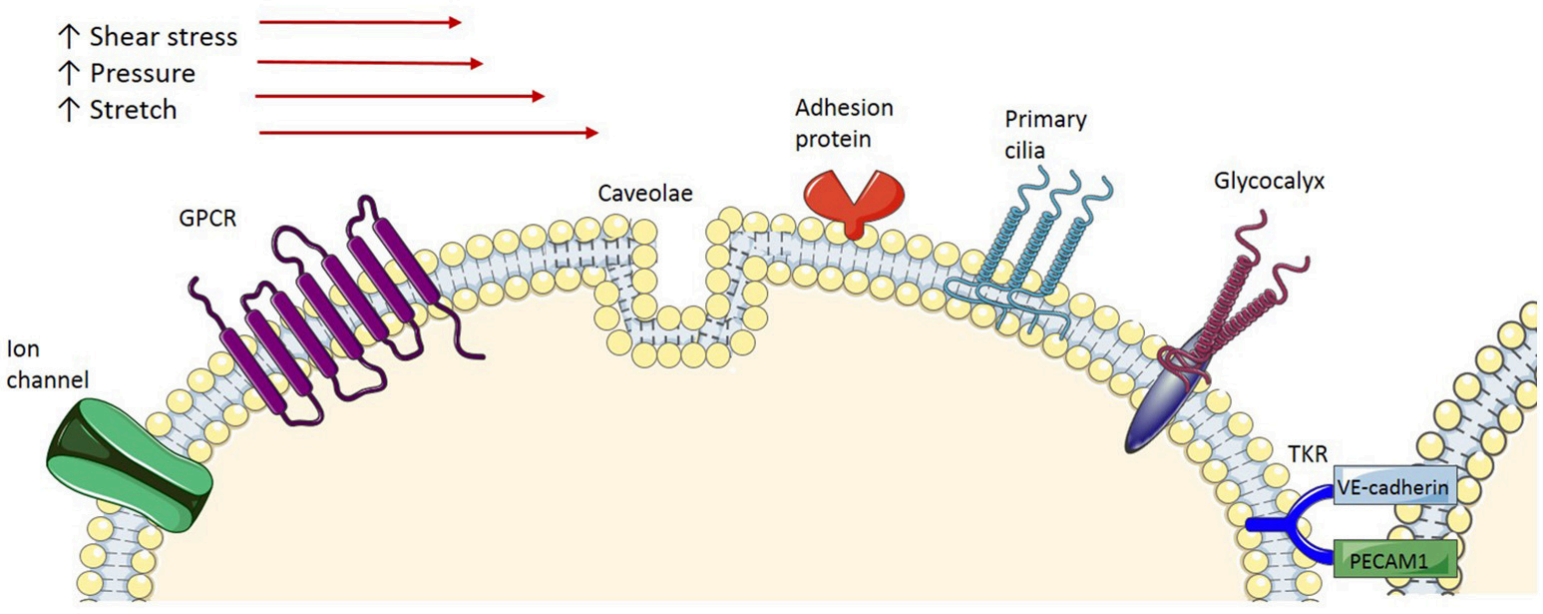
**Endothelial mechanoreceptors**. Ion channels: Various types of ion channels have been implicated in sensing shear stress. GPCR, G-protein-coupled receptors are demonstrated to be activated by shear stress; Caveola, Caveolae, membrane microdomains, are demonstrated to be involved in shear stress mechanotransduction; Adhesion proteins, Adhesion molecules expressed at the surface of the cells are exposed to shear stress and respond accordingly; Primary cilia, Protruding from the apical cell membrane, primary cilia sense, and signal the exterior shear stress; Glycocalyx, A transmembrane macromolecule, when exposed to shear stress undergoes a conformation change, which triggers signal transduction; TKR, Tyrosine kinase receptor is proposed to form a complex with integrin molecules to act as a mechanoreceptor.

As endothelial cells serve as a barrier between blood flow and vascular smooth muscle cells, it has been suggested that they act as mechanosensors that transform the mechanical stimuli into intracellular biochemical signals in order to cause changes in cell morphology, cell function and gene expression. Several membrane**-**associated complexes and cellular microdomains have been proposed as acting as mechanosensors, including ion channels, tyrosine kinase receptors (TKR), G**-**protein**-**coupled receptors (GPCR), adhesion proteins and structures such as caveolae, glycocalyx, primary cilia, and platelet endothelial cell adhesion molecule-1 (PECAM-1; Figure 2), which have been reviewed comprehensively in the literature (Tarbell et al., 2014; Zhou et al., 2014). Consequently, shear stress-induced activation of these signaling complexes results in transcriptional regulation of genes involved in maintaining homeostatic balance of processes such as endothelial cell proliferation, growth arrest, inflammatory and anti-inflammatory phenotypes and redox signaling. Of particular interest in this context are caveolae, which house many of the signaling proteins involved.

## Caveolae

**Figure 3:**
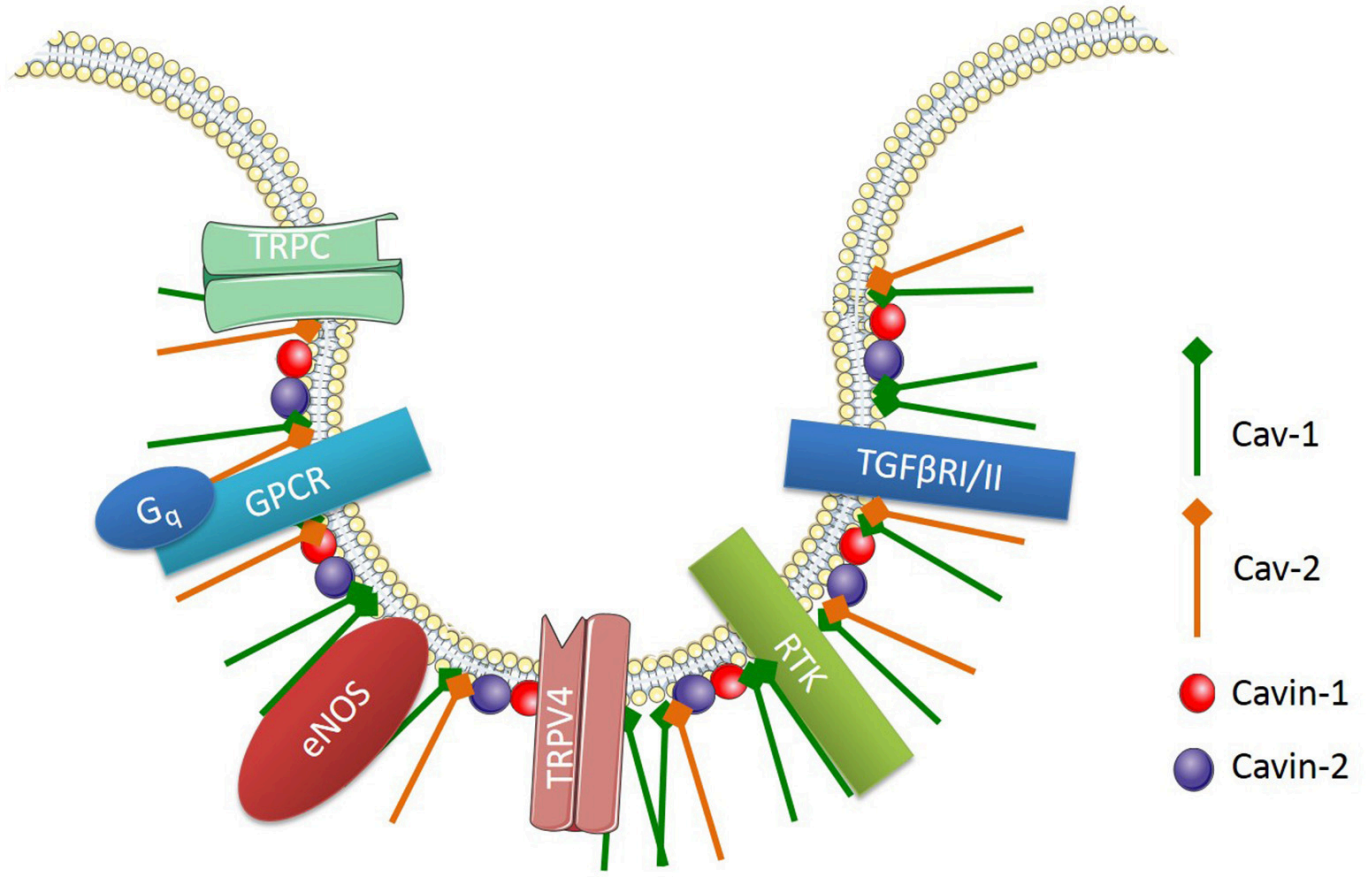
**Signaling proteins enriched in endothelial caveolae**. Cav-1, Caveolin-1; Cav-2, Caveolin 2; TRPC, transient receptor potential channel; GPCR, G-protein-coupled receptor; Gq, heterotrimeric G protein; TRPV4, TRP cation channel subfamily V number 4; RTK, receptor tyrosine kinase; TGFβRI/II, Transforming growth factor beta type I and II receptors.

Caveolae, flask-shaped non-coated structures on the cell surface, play a critical role in lipid homeostasis and signal transduction. Caveolae are predominantly described as 50- to 100-nm omega-shaped cell-surface invaginations (Yi et al., 2014). They are abundantly expressed on various cell types including vascular endothelial cells, epithelial cells, adipocytes, fibroblasts, and smooth muscle cells, but not on neuronal cells or lymphocytes (Quest et al., 2004). These membrane invaginations are enriched with various signaling proteins, which are involved in cell signaling pathways (Figure 3). One main difference between caveolae and lipid rafts is that caveolae contains a membrane protein known as caveolin (Cav).

There are three main isoforms of Cav, which include Cav-1, -2, and -3. Cav-1 and Cav-2 are highly expressed in non-muscle and smooth muscle cells, whereas Cav-3 is predominantly found in muscle cells (Murata et al., 2007). Endothelial cells express high levels of Cav-1, which is involved in endothelial inflammation, adhesion and phagocytosis. Importantly, Cav-1 expression is necessary for caveolae formation in non-muscle cells and it is involved in the regulation of numerous signaling cascades (Drab et al., 2001; Razani et al., 2001, 2002; Park et al., 2002). For example, endothelial Cav-1 regulates endothelial NO production, microvascular permeability and vascular remodeling (Murata et al., 2007). Along with caveolins, various supporting proteins have been identified as essential structures for the formation and function of caveolae. For instance, cavin-1 or polymerase transcript release factor (PTRF) and cavin-2 or serum deprivation protein response (SDPR) are both required for caveolae formation and function (Murata et al., 2007).

## eNOS and vascular reactivity

Caveolin-1 binds to endothelial nitric oxide (eNOS), inhibiting the activity of eNOS by limiting its accessibility to calcium-calmodulin (CaM) binding (Bernatchez et al., 2011) and thus preventing NO release (Trane et al., 2014). Mice deficient in eNOS lose vascular tone and are more susceptible to cardiovascular events (Knowles et al., 2000; Kuhlencordt et al., 2001). Indeed, Cav-1^−/−^ mice exhibit increased basal NO release and cyclic guanosine monophosphate (cGMP) production (Drab et al., 2001; Razani et al., 2001). Interestingly, Yu et al. (2006) demonstrated that Cav-1^−/−^ mice presented with reduced blood flow in response to vascular shear stress, but displayed no change in lumen diameter. They also showed an increase in vessel wall thickness and cellular proliferation compared to controls. The reconstitution of Cav-1 into the vessels ablated the response, suggesting that eNOS is unable to localize in the caveolae, resulting in impaired response to shear stress (Yu et al., 2006). Therefore, not only is Cav-1 important in the regulation of eNOS, but also its location in the caveolae is essential. Despite the increase in eNOS activity and NO production, and reports of reduced myogenic tone (Albinsson et al., 2007), Cav-1^−/−^ mice have no change in mean arterial blood pressure (Desjardins et al., 2008). This phenomenon has been suggested to be due to compensatory mechanisms from the long-term loss of Cav-1 or due to the plasticity of the vasculature (Insel and Patel, 2007). It was recently demonstrated that when Cav-1 was knocked down with cavnoxin, a peptide that targets the scaffolding domain of Cav-1 that binds to eNOS, vascular tone and blood pressure were reduced in control mice but not in Cav-1^−/−^ or eNOS^−/−^ mice (Bernatchez et al., 2011). Conversely, there have been reports of Cav-1^−/−^ mice with pulmonary hypertension, in which overactivity of eNOS leads to the reduction of protein kinase G (PKG) activity (Zhao et al., 2009). Interestingly, this reduction was abrogated in the double Cav-1^−/−^/eNOS^−/−^ mice, demonstrating the unique regulation of eNOS by Cav-1 (Zhao et al., 2009).

## Redox signaling

NADPH oxidase (Nox), known to be the predominant source of ROS, has seven isoforms; with Nox**-**1, **-**2, and **-**4 the most well-described in the vasculature (Ray and Shah, 2005). It has been demonstrated that Nox subunits may be preassembled and functional in caveolae (Yang and Rizzo, 2007). This study revealed, via western blotting, that Nox subunits are localized in the same light buoyant density membrane fractions as Cav-1 (Yang and Rizzo, 2007). Using both the crude method of depletion of membrane cholesterol with methyl-β-cyclodextrin (MβCD) and an isoluminol assay, they demonstrated that via TNFα or Ang II stimulation, ROS production was increased in raft membranes (Yang and Rizzo, 2007). Milovanova et al. (2008) have further shown that cessation of flow, a method of mimicking ischaemia, increased ROS production via Nox-2 in lungs and endothelial cells and that this was reduced in Cav-1^−/−^ mice. However, Cav-1^−/−^ mice also demonstrated similar production of ROS via Nox**-**2, compared to wild type mice following thrombin treatment (Milovanova et al., 2008). This suggests that in this setting, caveolae serve as a sensor of altered shear stress and that reduced Nox activity may be due to reduced membrane depolarization. Along with regulating eNOS, which is also a ROS generating enzyme, these studies suggest caveolae may also regulate ROS production through Nox.

## Caveolae and mechanosensation

Differing mechanical forces are shown to have varying impacts on caveolae. Laminar or physiological shear stress has been shown to increase caveolae number in cultured bovine aortic endothelial cells exposed to 10 dyn/cm^2^ for 1 h and 1 and 3 days at 19 dyn/cm^2^, compared to static conditions (Park et al., 1998; Boyd et al., 2003). Conversely, Sinha et al. (2011) showed that endothelial cells exposed to stretch via osmotic swelling demonstrate a significant reduction in the number of caveolae at the cell surface. Interestingly, they proposed that this was not due to an increase in endocytosis but rather a flattening and disassembly of caveolae (Sinha et al., 2011). Conversely, Albinsson et al. (2007) demonstrated that Cav-1 initiates downstream signaling in response to shear but not pressure or stretch. This was observed by reduced flow-induced dilation of myogenic tone in Cav-1 KO mice, but not with vessels constricted by activation of α1-adrenergic receptors and pressure (Albinsson et al., 2007). Perhaps in endothelial cells, caveolae act as mechanosensors to shear stress in order to elicit a cascade of events that promote NO production and vasodilation, but in response to stretch they instead elicit a structural response. As flattening is such an inherent property of caveolae, it has been proposed that this is a quick cell survival mechanism, creating a greater cell surface area (Parton and del Pozo, 2013). This is in line with other reports highlighting caveolae as membrane reservoirs (Dulhunty and Franzini-Armstrong, 1975; Sinha et al., 2011). It has been shown that upon flattening, caveolar scaffolding proteins are disassembled and released into the plasma membrane (caveolin) and cytosol (cavin) (Sinha et al., 2011). However, the purpose of such a disassembly is, aside from conformational changes, unclear. Whether this leads to compensatory signals to promote caveolae biogenesis due to their reduced number remain to be seen. It has been suggested that once caveolae disassemble, the protein subunits are able to exert actions on their own. This is supported by a study which demonstrated that dissociated cavin-1 could bind to a transcription factor known as binding factor of a type-I collagen promoter (BFCOL1) and in turn increase collagen type I, which is a major contributor to extracellular matrix (ECM) deposition (Hasegawa et al., 2000).

**Figure 4:**
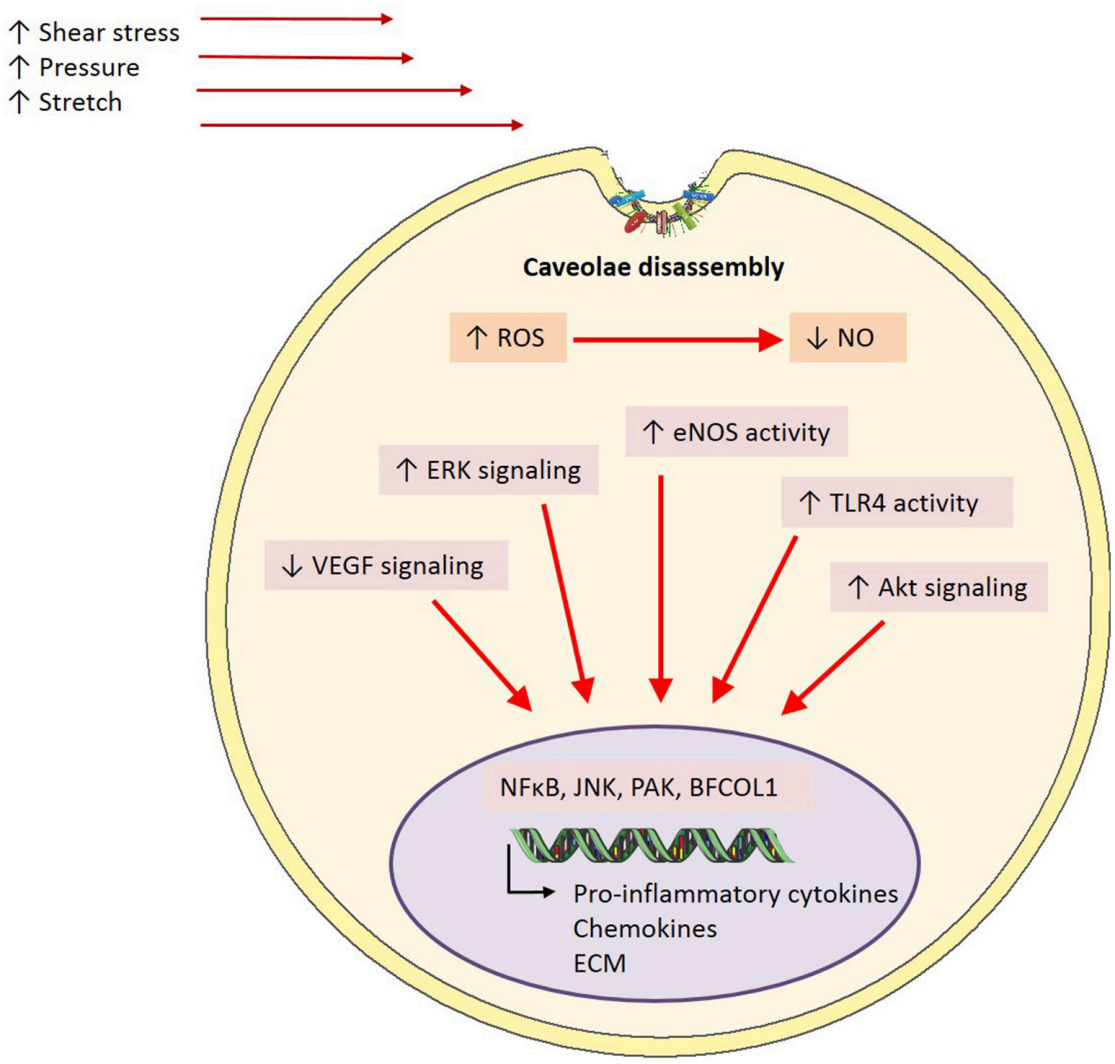
**Caveolae-mediated mechanisms in inflammation**. In the event of endothelial activation *via* inflammatory stimuli including shear stress, pressure or stretch, caveolae are proposed to play a mediatory role in downstream signaling pathways. Caveolae exert their effects through the inhibition of signaling cascades involved in the onset of inflammation through caveolin-1 directly interacting and binding to a number of signaling proteins including eNOS, TLR-4, and ERK. However, it is proposed that in disease states where there is a homeostatic imbalance in ROS:NO and subsequent endothelial activation, caveolae may disassemble, and in turn uncouple from signaling proteins. This results in upregulated activity and phosphorylation of signaling proteins key in inflammation, and thus enhance pro-inflammation transcriptional activity (ROS, reactive oxygen species; NO, nitric oxide; eNOS, endothelial nitric oxide synthase; ERK, extracellular signal-regulated kinases; TLR4, toll-like receptor 4; NFκB, nuclear factor kappa B; JNK, c-Jun N-terminal kinase; PAK, p21-activated kinase; BFCOL1, binding factor of a type-I collagen promoter; ECM, extracellular matrix).

As well as being able to form homo-oligomers, Cav-1 is able to interact with various other intracellular signaling molecules enriched in caveolae including G protein-coupled receptors, tyrosine kinases, eNOS and some components of the MAPK pathway (Li et al., 1995; García-Cardeña et al., 1996; Liu et al., 1996, 1997; Huang et al., 1997; Engelman et al., 1998). Several reports in literature have implicated these molecules as major players in shear stress-mediated activation of endothelial cells. Studies exposing cells to chronic shear stress have demonstrated increased tyrosine phosphorylation and activation of surface proteins involved in shear-sensitive signaling pathways, such as Akt, eNOS, and ERK (Figure 4) (Sun et al., 2002, 2003; Boyd et al., 2003; Rizzo et al., 2003). Further, studies involving cholesterol depletion using β-cyclodextrin (which disrupts caveolar structure) or cyclosporin A (which depletes cholesterol from caveolae without affecting its structure), have shown inhibited flow activation of ERK and reduced phosphorylation of eNOS, respectively (Park et al., 1998; Lungu et al., 2004). The suspected role of caveolae in mechanotransduction is further demonstrated by diminished VEGF signaling, which is critical in both shear stress signaling pathways and eNOS phosphorylation, in the setting of Cav-1 deficiency (Wang et al., 2002; Sonveaux et al., 2004; Jin et al., 2005). Moreover, a study by Yu et al. (2006) demonstrated for the first time in an *in vivo* setting the role for caveolae as mechanotransducers and Cav-1 in arterial responses to changes in blood flow. They showed impaired flow-dependent arterial remodeling, defects in flow-induced vasodilation and blunted eNOS activation in Cav-1^−/−^ mice, which were all rescued with the reconstitution of endothelial specific Cav-1 (Yu et al., 2006). Furthermore, a reduction in basal eNOS phosphorylation on serine 1176, a key regulatory site of phosphorylation by various shear-sensitive kinases, seen in Cav-1^−/−^ mouse vessels was returned back to wild-type levels with the restoration of endothelial caveolae (Yu et al., 2006). It has recently been suggested that in pulmonary microvascular endothelial cells PECAM-1 and caveolae form a mechanosensing complex that is activated with cessation of shear (Noel et al., 2013).

In addition to their role as mechanosensors, the effects of caveolae, or specifically Cav-1 on inflammatory signaling also has important functional consequences. For example, in Cav-1^−/−^ mice on an apoE^−/−^ background, mice have reduced plaque size (Frank et al., 2004) and when only endothelial caveolin-1 is restored, this protective phenotype is lost (Fernández-Hernando et al., 2009). Intravital studies with these mice also demonstrated that these effects are most likely due to the reduced leukocyte adhesion due to diminished levels of MCP-1 and VCAM-1 (Engel et al., 2011). It is thought that interactions of Cav-1 with toll-like receptor 4 (TLR4), eNOS, and MAPK underlies many of these effects (Figure 4). Indeed, phosphorylation of Cav-1 after a LPS insult results in TLR4 activation, NFκB and cytokine production in lung endothelial cells (Jiao et al., 2013). Collectively, these studies indicate an important role for caveolae/Cav-1 in haemodynamic mechanotransduction and the resulting inflammation, based on their involvement in key shear-induced intracellular signaling pathways.

## Summary and unanswered questions

The effects of haemodynamic stress on vascular endothelium have been characterized in literature, with a strong focus on the cellular mechanisms involved more recently. However, there still remains a gap in the understanding of how the initial interaction between the endothelium and shear stress occurs. Candidates for shear stress sensors have been proposed including caveolae, yet there is still a large gap in knowledge regarding mechanisms of endothelial transduction. Although we have shown the central role that caveolae play in mediating shear stress-induced signaling, it is of importance to consider other structures commonly associated with mechanosensation and to more importantly understand the interplay between the various mechanosensors in both physiological and pathophysiological contexts. It would be of interest to see whether the effects observed in caveolae-deficient settings are actually due to the absence of caveolae or due to specific intracellular signaling molecules. Further, the influence of the differential shear patterns on the endothelium and its interaction with caveolae should not be discounted. Thus, further studies are vital for the elucidation of the mechanisms underlying the mechanotransduction of shear stress and the role of endothelial mechanosensors such as caveolae in cardiovascular homeostasis.

## Author contributions

WS and DM drafted the manuscript and contributed to the interpretation of data in the review. WS prepared the manuscript and figures. WS, DM, KA, and JC-D reviewed, edited, and approved the final version of the manuscript.

### Conflict of interest statement

The authors declare that the research was conducted in the absence of any commercial or financial relationships that could be construed as a potential conflict of interest.
